# Cytosolic phospholipase A2 links tau pathology to insulin signaling impairment in Alzheimer’s disease

**DOI:** 10.3389/fnagi.2025.1671986

**Published:** 2025-11-07

**Authors:** Faruk Hossen, Javier Hung, Hamza Odeh, Grace Y. Sun, James C. Lee

**Affiliations:** 1 Richard and Loan Hill Department of Biomedical Engineering, University of Illinois at Chicago, Chicago, IL, United States; 2 Department of Biochemistry, University of Missouri, Columbia, MO, United States

**Keywords:** oligomeric tau, insulin signaling, cerebral endothelial cells, cPLA2, Alzheimer’s disease, IRS-1, Caveolin-1

## Abstract

Although impaired insulin signaling in the brain has been recognized as a key factor in the development and progression of Alzheimer’s disease (AD), the underlying mechanisms remain incompletely understood. Given that overactivation of cytosolic phospholipase A2 (cPLA2) has been implicated in AD, we tested the hypothesis that oligomeric tau (oTau) activates cPLA2, which in turn negatively affects Caveolin-1 (Cav-1) and insulin signaling. In the cerebral cortex and hippocampus of 12-months-old 3xTg-AD mice, we observed an upregulation of phosphorylated cPLA2 (p-cPLA2), accompanied by downregulation of Cav-1 and impaired insulin signaling. Specifically, we found significant decreases in insulin receptor-α (IR-α) and insulin receptor-β (IR-β) expression, along with increased levels of phospho-insulin receptor substrate 1 at Ser307 [p-IRS-1 (Ser307)] and decreased levels of p-IRS-1 (Tyr895), compared to wild-type (WT) mice. To further investigate the role of cPLA2 in insulin signaling impairment in AD, we demonstrated that oTau activated cPLA2 in primary mouse cerebral endothelial cells (CECs), leading to Cav-1 downregulation and disrupted insulin signaling. Notably, these detrimental effects of oTau on Cav-1 and insulin signaling were abolished when cPLA2 expression was depleted using small interfering RNA (siRNA). In conclusion, our study highlights the pivotal role of cPLA2 in regulating Cav-1 function and insulin signaling in AD, offering insights into potential therapeutic targets for mitigating insulin resistance associated with the disease.

## Introduction

Alzheimer’s disease (AD) is a progressive neurodegenerative disorder characterized by cognitive decline, synaptic dysfunction, and neuronal loss (Serrano-Pozo et al., 2011; Tenchov et al., 2024; Testo et al., 2024). Recently, a strong bidirectional relationship has been reported between AD and type 2 diabetes mellitus (T2DM), with insulin resistance and dysregulated glucose metabolism as common features of both conditions (Chatterjee and Mudher, 2018; Chornenkyy et al., 2019; Kshirsagar et al., 2021). Growing evidence indicates that disruptions in insulin receptor (IR) signaling contribute to AD pathology (Plum et al., 2005; Huang et al., 2010; Pomytkin et al., 2018), as insulin plays a fundamental role in neuronal survival, synaptic plasticity, and energy metabolism (Gerozissis, 2008; Huang et al., 2010; Milstein and Ferris, 2021). Mechanisms such as hyperphosphorylation of insulin receptor substrate-1 (IRS-1), oxidative stress, chronic neuroinflammation, and amyloid-β/tau-mediated toxicity have been implicated in impairing insulin signaling in the AD brain (Boura-Halfon and Zick, 2009; Rains and Jain, 2011; Copps and White, 2012; El Khoury et al., 2014; Cheignon et al., 2018; Gali et al., 2019; Gonçalves et al., 2019; Zheng and Wang, 2021). Importantly, abnormal tau has recently been found to potentiate the toxic environment by interfering with the insulin signaling cascade in the brains of patients with AD (El Idrissi and Alonso, 2022), highlighting the deleterious consequences of tau pathology-induced insulin resistance in the brain (Gonçalves et al., 2019). However, the precise cellular pathways linking tau pathology to insulin resistance remain unclear, which requires further investigation.

Abnormal cytosolic phospholipase A2 (cPLA2) activity in the brain has been implicated in AD (Stephenson et al., 1996; Gynther et al., 2022). cPLA2 is a lipid-metabolizing enzyme that hydrolyzes membrane phospholipids, releasing arachidonic acid and lysophospholipids (Leslie, 2015). These bioactive lipid mediators contribute to cellular homeostasis, and neuroinflammation and oxidative stress when dysregulated (Kramer et al., 1996; Sun et al., 2014). In recent years, there has been growing interest in understanding the role of cPLA2 in regulating cellular functions under both physiological and pathological conditions (Hirabayashi et al., 2004; Murakami et al., 2011; Leslie, 2015). A notable feature of cPLA2 is its association with cell surface receptors that activate signaling pathways linked to protein kinases and the production of reactive oxygen species (ROS) (Sun et al., 2014). In the central nervous system (CNS), cPLA2 activation has been implicated in processes such as neuronal excitation, synaptic secretion, apoptosis, cell-cell interactions, cognitive and behavioral functions, oxidative stress, and inflammatory responses, all of which contribute to the pathogenesis of several neurodegenerative diseases including AD (Kriem et al., 2005; Sanchez-Mejia et al., 2008; Sun et al., 2010, 2014; Hegde et al., 2023). In AD, aberrant activation of cPLA2 has been linked to neuronal dysfunction, synaptic deficits, and blood-brain barrier (BBB) disruption (Stephenson et al., 1996; Kerman et al., 2022; Hegde et al., 2023). Importantly, cPLA2-driven lipid modifications alter cellular membranes and their compartments, including lipid rafts–specialized microdomains essential for signal transduction (Simons and Toomre, 2000; Mrówczyñska, 2012; Boi et al., 2022). Given the role of lipid rafts in supporting IR localization and function (Czech, 2000), it is plausible that cPLA2 overactivation may negatively impact insulin signaling in the AD brain.

Caveolin-1 (Cav-1), a structural protein of lipid rafts, plays a critical role in regulating insulin receptor trafficking and signal transduction (Cohen et al., 2003a; Haddad et al., 2020; Peng et al., 2021). Reduced Cav-1 expression has been associated with endothelial dysfunction and impaired insulin signaling in neurodegenerative diseases (Cohen et al., 2003b; Shetti et al., 2023). Although cPLA2-mediated lipid alterations are known to disrupt membrane microdomains, how aberrant cPLA2 activity affects Cav-1 and insulin signaling remains an open question.

In this study, we test the hypothesis that oligomeric tau (oTau) activates cPLA2, leading to Cav-1 downregulation and impaired insulin signaling. In both *in vivo* and *in vitro* experiments, we demonstrate that cPLA2 activation is associated with reduced Cav-1 expression and disrupted insulin signaling in the brain of 3xTg mouse and in primary mouse cerebral endothelial cells (CECs). Notably, knockdown of cPLA2 using small interfering RNA (siRNA) counteracts the ill effects of oTau on insulin signaling in CECs by restoring the Cav-1 expression level, highlighting the pivotal role of cPLA2 activation in oTau-induced insulin signaling impairment in AD through its negative effect on Cav-1 expression.

## Materials and methods

### Materials

Tau-441 from rPeptide (Bogart, GA), and primary antibodies and reagents were purchased as follows: Anti-tau (T22) from Millipore Sigma (St. Louis, MO); antibodies against IR-α, IR-β, IRS-1, p-IRS-1 (Ser307), p-IRS-1 (Tyr895), cPLA2, p-cPLA2 and Cav-1 from Cell Signaling (Beverly, MA); dimethyl sulfoxide (DMSO), cOmplete protease inhibitor cocktail, PhosSTOP phosphatase inhibitor cocktail, β-mercaptoethanol (β-ME), Lipofectamine 3000 transfection reagent and cPLA2 siRNA from Thermo Fisher Scientific (Waltham, MA); Ham’s F-12 Nutrient Mix from Crystalgen Inc., (Commack, NY); EGM-2 MV BulletKit from Lonza (Walkersville, MD); penicillin/streptomycin (P/S), Hanks’ Balanced Salt Solution (HBSS), and Dulbecco’s phosphate-buffered saline (DPBS) from Life Technologies (Grand Island, NY); fibronectin, collagen from calf skin, laminin, and Minimum Essential Media Eagle (MEM) from Millipore Sigma (St. Louis, MO); papain and DNase from Worthington Biochemical (Lakewood, NJ); radio-immunoprecipitation assay (RIPA) buffer, BCA protein assay kit, SuperSignal™ West Femto maximum sensitivity substrate, SuperSignal™ West Pico Plus chemiluminescent substrate, and Restore™ PLUS western-blot stripping buffer from Thermo Fisher Scientific (Waltham, MA); and Laemmli sample buffer and tris-buffered saline (TBS) from Bio-Rad (Hercules, CA). Antibodies, key reagents, and assay kits used in this study, along with their detailed information (company name, catalog number, etc.), are provided in Supplementary Tables 1, 2.

### Oligomeric tau preparation and characterization

We prepared oligomeric tau following the protocol outlined in Majerova et al. (2019) and Hossen et al. (2024b). Briefly, lyophilized powder of tau-441 was resuspended at a concentration of 1 mg/mL in deionized water. Oligomerization of tau was induced by adding 0.2% heparin in PBS, followed by incubation for 3 h at 37 °C in a CO_2_ incubator. oTau was characterized by Western blot analysis and atomic force microscopy (AFM) imaging (Supplementary Figure 1).

### Animals

C57BL/6 mice and 3xTg AD mice were purchased from Jackson Laboratory (Bar Harbor, Maine). These mice were maintained under a 12-h light/dark cycle and temperature-controlled conditions, with *ad libitum* access to food and water. All experiments were performed in accordance with the approved animal protocols and guidelines established by the Institutional Animal Care and Use Committee (IACUC) at the University of Illinois Chicago (UIC).

### Isolation, culture, and treatment of primary mouse CECs

Cerebral endothelial cells were isolated from C57BL/6 mice as described previously (Marottoli et al., 2021). Detailed methodology and cell characterization can be found in our previous studies (Hossen et al., 2024a, b). To investigate the effects of oTau on CECs, cells were serum-starved in EBM-2 Endothelial Basal Media for 3 h. Subsequently, they were treated with oTau (0.2 μM) for either 30 min or 24 h, depending on the specific characterizations. To investigate whether the cPLA2 signaling pathway was involved in oTau-induced alterations of insulin signaling in CECs, cPLA2 gene silencing was achieved through siRNA transfection, following the instructions provided by the commercially available Lipofectamine 3000 transfection reagents.

### cPLA2 gene silencing by siRNA transfection using lipofectamine 3000 reagent

Cytosolic phospholipase A2 gene silencing was performed using siRNA transfection with the Lipofectamine 3000 transfection reagent, following the manufacturer’s protocol. Briefly, cells were transfected with cPLA2-targeting siRNA. The siRNA and lipofectamine 3000 transfection reagent were mixed in Opti-MEM medium to form transfection complexes, which were then added to cells cultured in antibiotic-free medium. After 48 h of incubation, the efficiency of cPLA2 silencing was confirmed by Western blot analysis. Detailed information of the siRNA targeting cPLA2 is provided in the Supplementary file.

### Mouse brain extraction and homogenate preparation for western blot

Mice at the age of 12-months old were anesthetized with CO2, and brains were carefully dissected. The cerebral cortex and hippocampus were separated from the brain. Tissue (cerebral cortex and hippocampus) was either processed immediately or flash-frozen in liquid nitrogen and stored at −80 °C until further use. Fresh or frozen tissue was mechanically homogenized in ice-cold RIPA lysis buffer supplemented with complete™ protease inhibitor cocktail and phosphatase inhibitor cocktail, using approximately 700 μL lysis buffer per 100 mg tissue. Homogenization was performed with a tissue homogenizer on ice. Homogenates were transferred to 1.5 mL microcentrifuge tubes and centrifuged at 14,000 × *g* for 20 min at 4 °C. The resulting supernatant was collected into clean tubes and stored at −80 °C until further use for Western blot analysis.

### Western blotting analysis

Cells were cultured in 35 mm dishes until 80%–90% confluence. Cells were treated with oTau (0.2 μM) for 30 min or 24 h, depending on the specific characterizations. For the assessment of phosphorylated proteins, a 30-min incubation was sufficient. After treatment, cells were washed with PBS and lysed with ice-cold RIPA buffer supplemented with a cocktail of protease and phosphatase inhibitors for 30 min at 4 °C with gentle agitation. Cell lysates were collected into 1.5 mL Eppendorf tubes using a cell scraper and centrifuged at 13,000 rpm for 20 min at 4 °C. After centrifugation, the supernatants were collected, and the protein concentration in the cell lysate was determined using a BCA assay. Western blot analysis of all targeted proteins related to insulin signaling was carried out as previously described (Hossen et al., 2024a). Each experiment was repeated at least three times. In the experiment for detection of IR-α, IR-β, and Cav-1, cells were treated with oTau (0.2 μM) for 24 h. The membrane was reblotted with antibodies for β-actin as a protein loading control. However, in experiments in which a short incubation time (30 min) was used to detect phosphorylation of proteins of interests, we observed consistency in the loading of the total proteins of interest. Therefore, the total proteins of interest also served as loading control. Western blot images were analyzed using Image Studio Lite version 5.2 software. Each experimental group consisted of *n* = 6 animals (unless otherwise specified anywhere).

### Data analysis

For most experiments, cells were cultured under the conditions stated above, and measurements were conducted in triplicate for each experimental group. The results are expressed as the mean ± standard deviation (SD). Statistical analysis involving multiple groups was performed using one-way analysis of variance (ANOVA), followed by Tukey’s *post hoc* honestly significant difference (HSD) test, as executed in GraphPad Prism (version 8.10). Statistical significance was defined as *p*-values < 0.05.

## Results

### Increased phosphorylation of cPLA2 in 3xTg AD mouse brains

**FIGURE 1:**
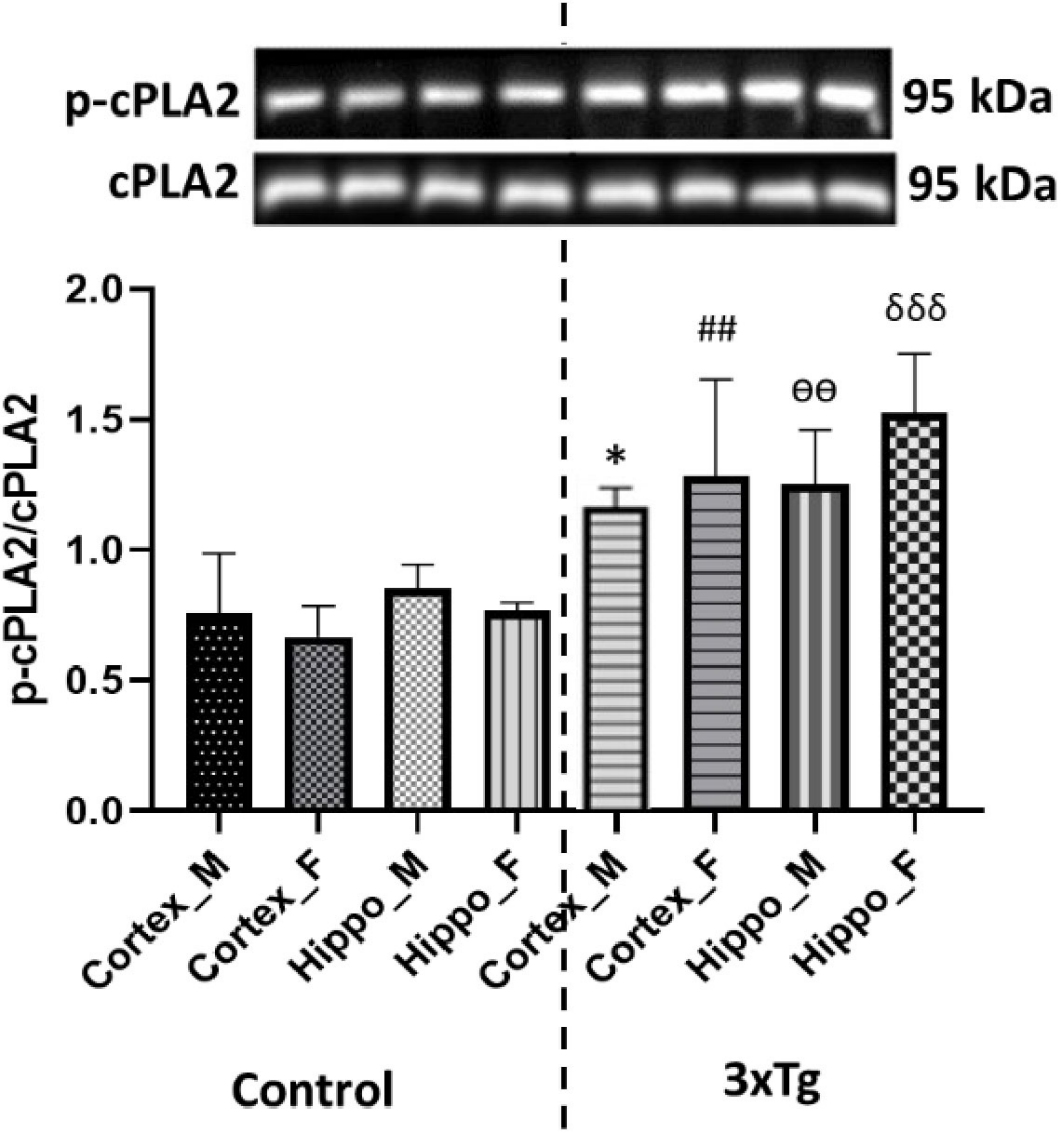
Phospho-cPLA2 expression is significantly increased in both the cortex and hippocampus of 3xTg AD mouse brains compared to control mouse brains. Data are presented as mean ± SD. Statistical significance is denoted as follows: *p < 0.05 compared to the control male mouse cortex, ^##^*p* < 0.01 compared to the control female mouse cortex, ^ѳѳ^*p* < 0.001 compared to the control male mouse hippocampus, and ^δδδ^*p* < 0.001 compared to the control female mouse hippocampus.

Cytosolic phospholipase A2 activation has been implicated in oxidative stress and inflammatory responses, which underlie the pathogenesis of several neurodegenerative diseases (Sun et al., 2014). It also plays a role in modifying cellular membrane composition and physical characteristics, altering cell, memory, and cognitive function (Hegde et al., 2023). To investigate cPLA2 activation in the cerebral cortex and hippocampus of C57BL/6 and 3xTg AD mouse brains, we performed western blot analysis for phosphorylated cPLA2 in brain tissue. Our results showed that phosphorylation of cPLA2 was significantly increased in both the cerebral cortex and hippocampus of 3xTg AD mice compared to control mice (Figure 1), suggesting that cPLA2 overactivation in 3xTg AD mouse brains may be partly in response to the AD pathology.

### Downregulation of Caveolin-1 in 3xTg AD mouse brains

**FIGURE 2:**
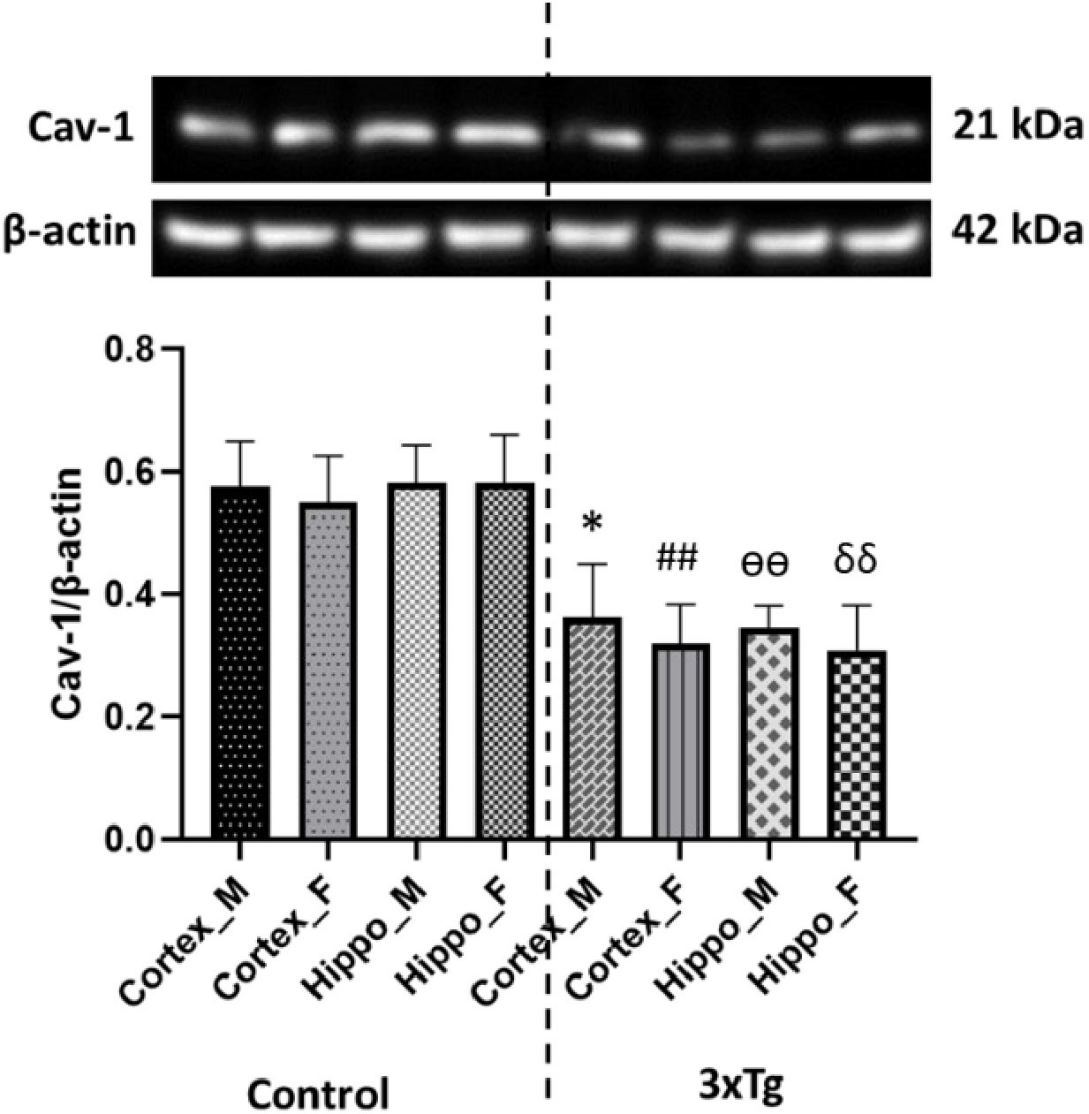
Caveolin-1 (Cav-1) expression is significantly decreased in both the cortex and hippocampus of 3xTg AD mice compared to control mice. Data are presented as mean ± SD. Statistical significance is denoted as follows: *p < 0.05 compared to the control male mouse cortex, ^##^*p* < 0.01 compared to the control female mouse cortex, ^ѳѳ^*p* < 0.01 compared to the control male mouse hippocampus, and ^δδ^*p* < 0.01 compared to the control female mouse hippocampus.

Caveolin-1, a functional protein of membrane lipid rafts, plays a critical role in insulin signaling by interacting with the insulin receptor (Nystrom et al., 1999; Haddad et al., 2020). Cav-1 depletion has been extensively implicated in the pathogenesis of type 2 diabetes (Cohen et al., 2003b). Importantly, Cav-1 knockout mice develop insulin resistance (David et al., 2012). To investigate Cav-1 expression in the cerebral cortex and hippocampus of C57BL/6 and 3xTg AD mouse brains, we performed western blot analysis. Our results revealed that Cav-1 expression was significantly reduced in both the cerebral cortex and hippocampus of 3xTg AD mouse brains compared to control mouse brains (Figure 2).

### Disrupted insulin signaling in the cerebral cortex and hippocampus of 3xTg AD mice

Insulin receptor is present throughout the brain and serves important functions in whole-body metabolism and brain function (Plum et al., 2005; Milstein and Ferris, 2021). Although the insulin receptor is found ubiquitously throughout the brain, its expression is higher in the cortex and hippocampus (Hopkins and Williams, 1997). IRS-1 plays an important role in regulating insulin signaling through the phosphorylation of its serine and tyrosine residues (Boura-Halfon and Zick, 2009; Copps and White, 2012). Studies show that phosphorylation of serine residues in IRS-1 inhibits insulin signaling, whereas phosphorylation of tyrosine residues promotes insulin signaling (Aguirre et al., 2002; Werner et al., 2004; Gual et al., 2005).

**FIGURE 3:**
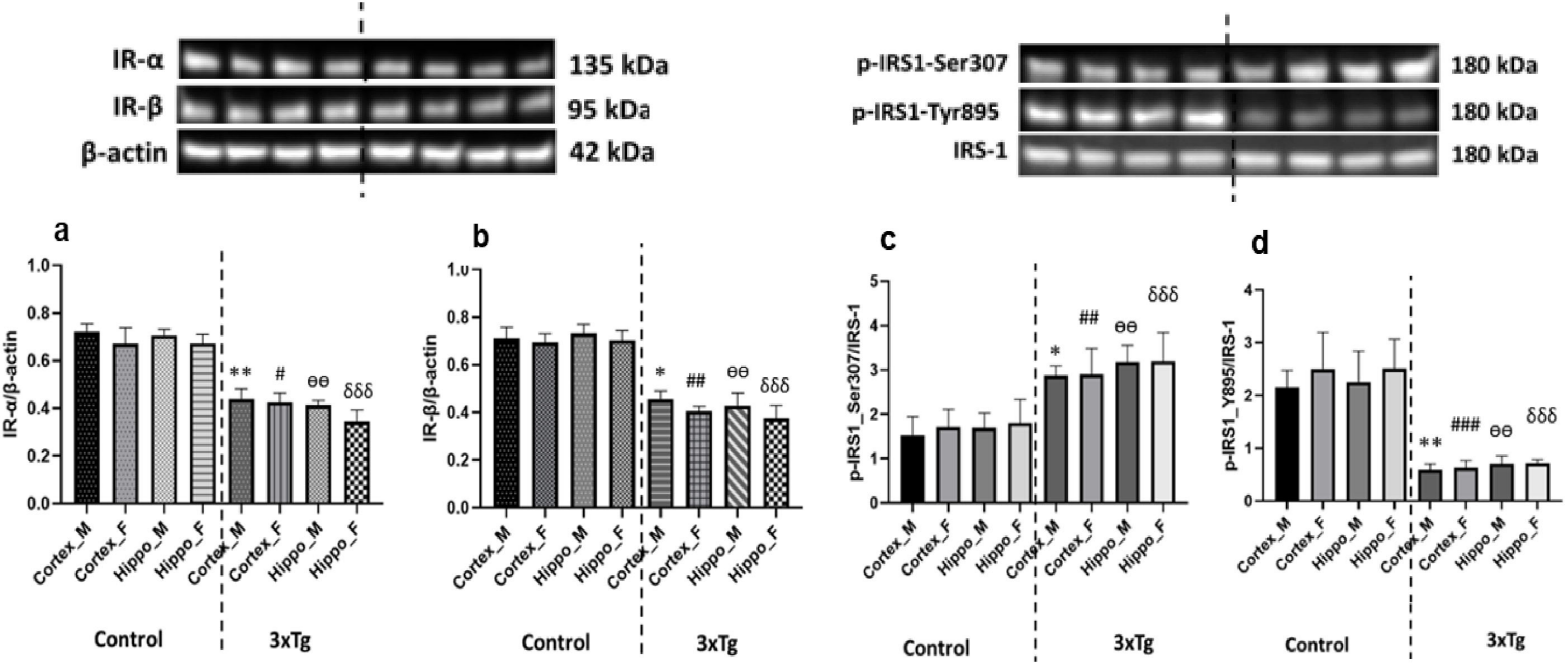
**(a)** Insulin receptor-α (IR-α) and **(b)** IR-β expression levels are significantly decreased in both the cortex and hippocampus of 3xTg AD mouse brains compared to control mouse brains. **(c)** Phosphorylation at the inhibitory Ser307 site of IRS-1 is significantly increased, while **(d)** phosphorylation at the activating Tyr895 site of IRS-1 is significantly decreased in the cortex and hippocampus of 3xTg AD mice compared to controls. Data are presented as mean ± SD. Statistical significance is denoted as follows: *p < 0.05, **p < 0.01 compared to the control male mouse cortex; #*p* < 0.05, ##*p* < 0.01, ###*p* < 0.001 compared to the control female mouse cortex; ^ѳѳ^*p* < 0.001 compared to the control male mouse hippocampus; δδδ*p* < 0.001 compared to the control female mouse hippocampus.

To investigate insulin signaling alterations in AD mice brain, we analyzed the expression of IR subunits (IR-α and IR-β) and the phosphorylation of IRS-1 in the cerebral cortex and hippocampus of C57BL/6 and 3xTg AD mice. Western blot analysis revealed a significant reduction in the expression of both IR-α and IR-β subunits in the 3xTg AD mouse brains compared to controls (Figures 3a, b), suggesting compromised insulin receptor availability. Additionally, we observed a marked increase in IRS-1 phosphorylation at the inhibitory Ser307 residue and a concurrent decrease at the activating Tyr895 site in 3xTg AD mice (Figures 3c, d). These findings collectively indicate an impairment in insulin receptor expression and signaling within these brain regions of AD mice.

### siRNA mediated silencing of cPLA2 gene in CECs

**FIGURE 4:**
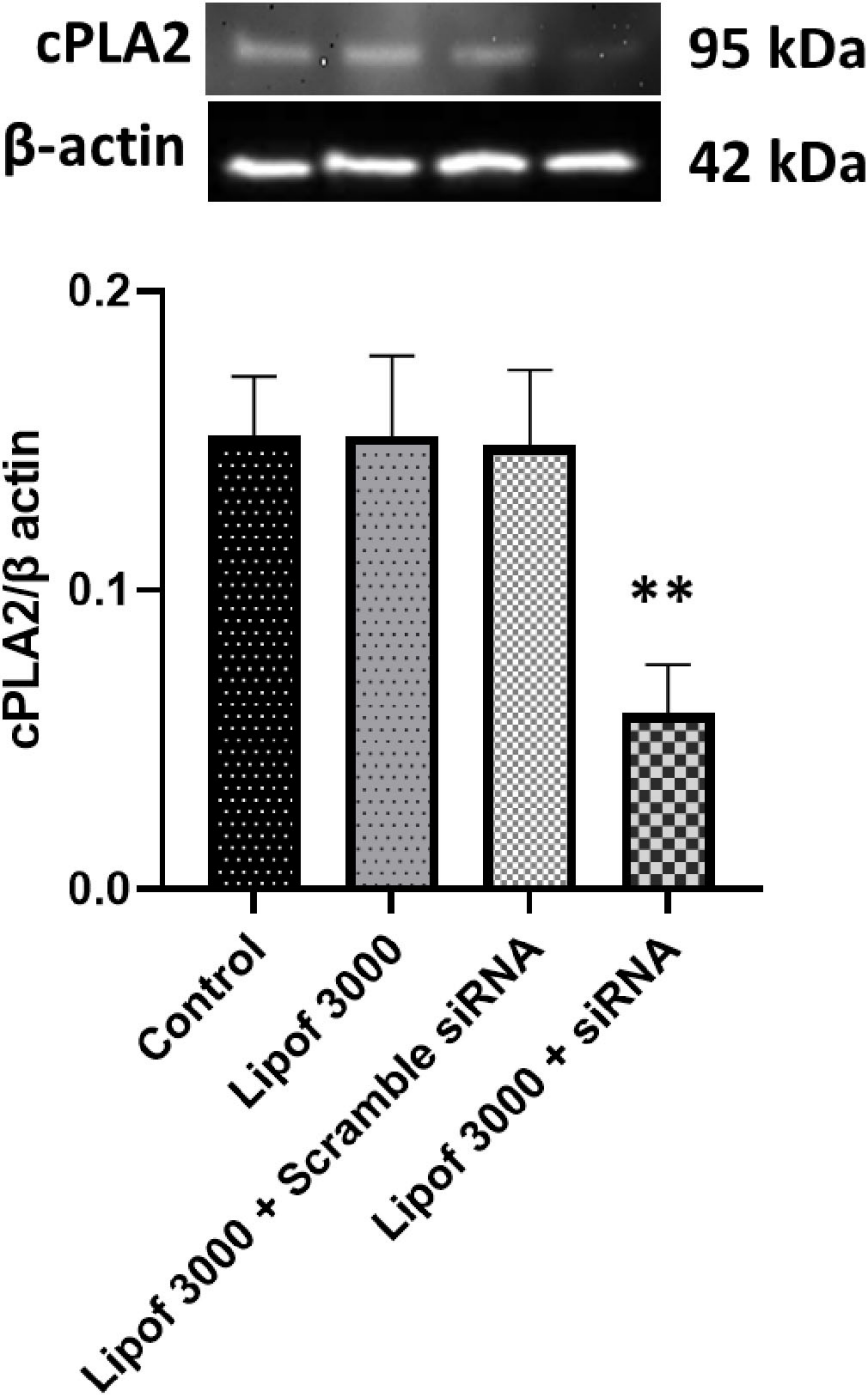
Cytosolic phospholipase A2 (cPLA2) expression was significantly reduced following siRNA transfection compared to the control. cPLA2 was depleted by ∼70%. Data are presented as mean ± SD. Statistical significance is denoted as follows: **p < 0.01, compared to the control.

To examine whether cPLA2 activation and insulin signaling impairment are causally related, we used siRNA transfection to silence cPLA2 gene expression in endothelial cells (CECs). Transfection with cPLA2 siRNA using Lipofectamine 3000 significantly reduced cPLA2 expression by approximately 70% compared to the control groups (Figure 4). Treatment with the transfection reagent (Lipofectamine 3000) alone or scramble siRNA was used as a control and had no effect on cPLA2 expression (Figure 4).

### Silencing cPLA2 restores oTau-induced downregulation of Cav-1 in CECs

**FIGURE 5:**
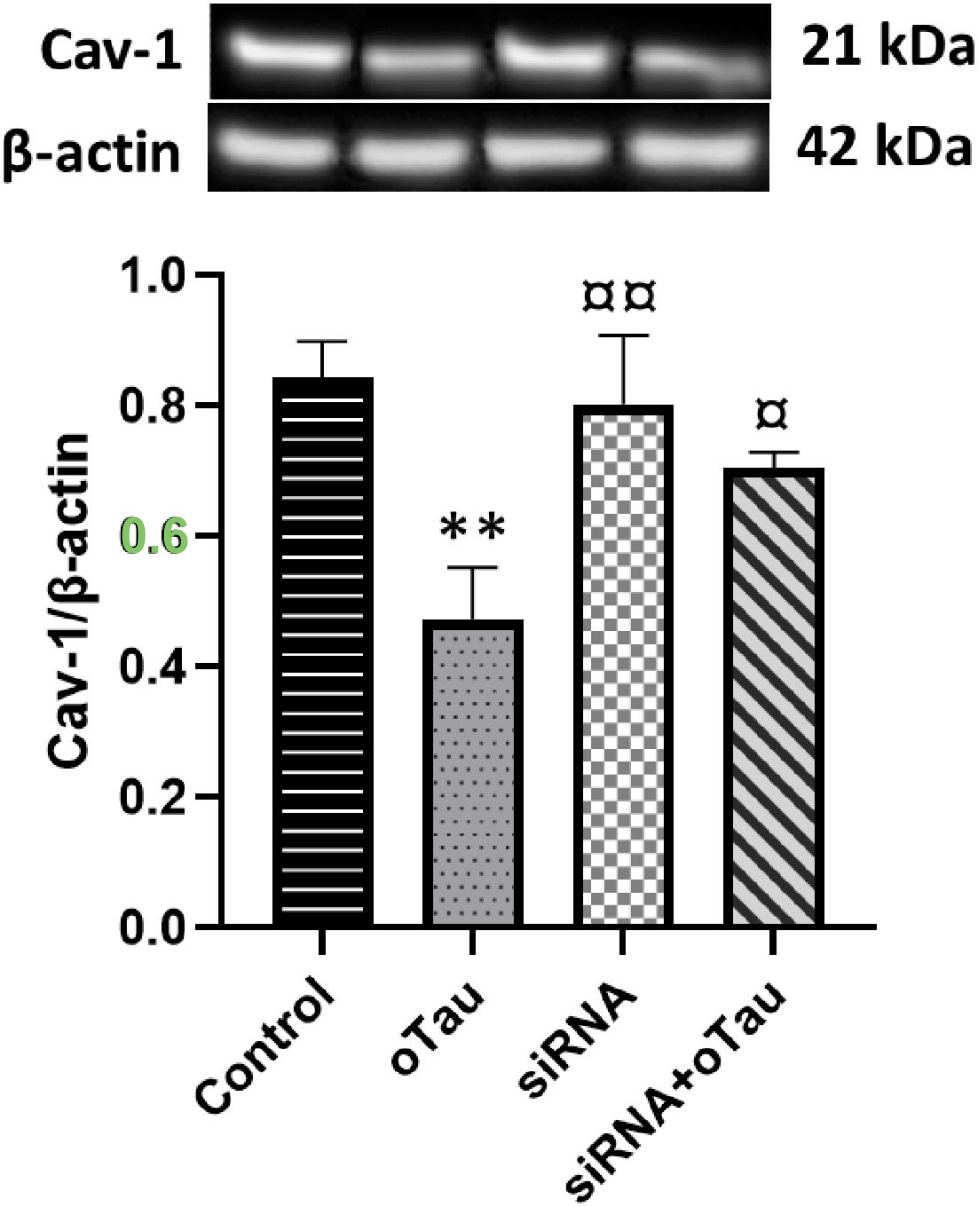
Caveolin-1 (Cav-1) expression is significantly decreased in the oTau-treated group compared to the control group. However, silencing of cPLA2 gene restores Cav-1 expression in oTau-exposed CECs. Data are presented as mean ± SD. Statistical significance is denoted as follows: **p < 0.01, compared to the control, ^¤^*p* < 0.05, ^¤¤^*p* < 0.01 compared to the oTau treated group.

Caveolin-1 is essential for improving insulin sensitivity, and studies have shown that Cav-1-deficient mice exhibit insulin resistance and defective insulin receptor protein expression (Cohen et al., 2003b; Peng et al., 2021). Several studies have reported a link between tau pathology and impaired insulin signaling in the AD brain (Gonçalves et al., 2019; El Idrissi and Alonso, 2022; Ennis et al., 2023); however, the underlying mechanism remains unknown. We examine the role of cPLA2 in the effect of oTau on Cav-1 expression. As shown in Figure 5, oTau significantly reduced Cav-1 expression in CECs. However, this oTau-induced downregulation of Cav-1 was reversed when cPLA2 expression was silenced by siRNA transfection.

### Silencing cPLA2 restores insulin signaling disrupted by oTau in CECs

**FIGURE 6:**
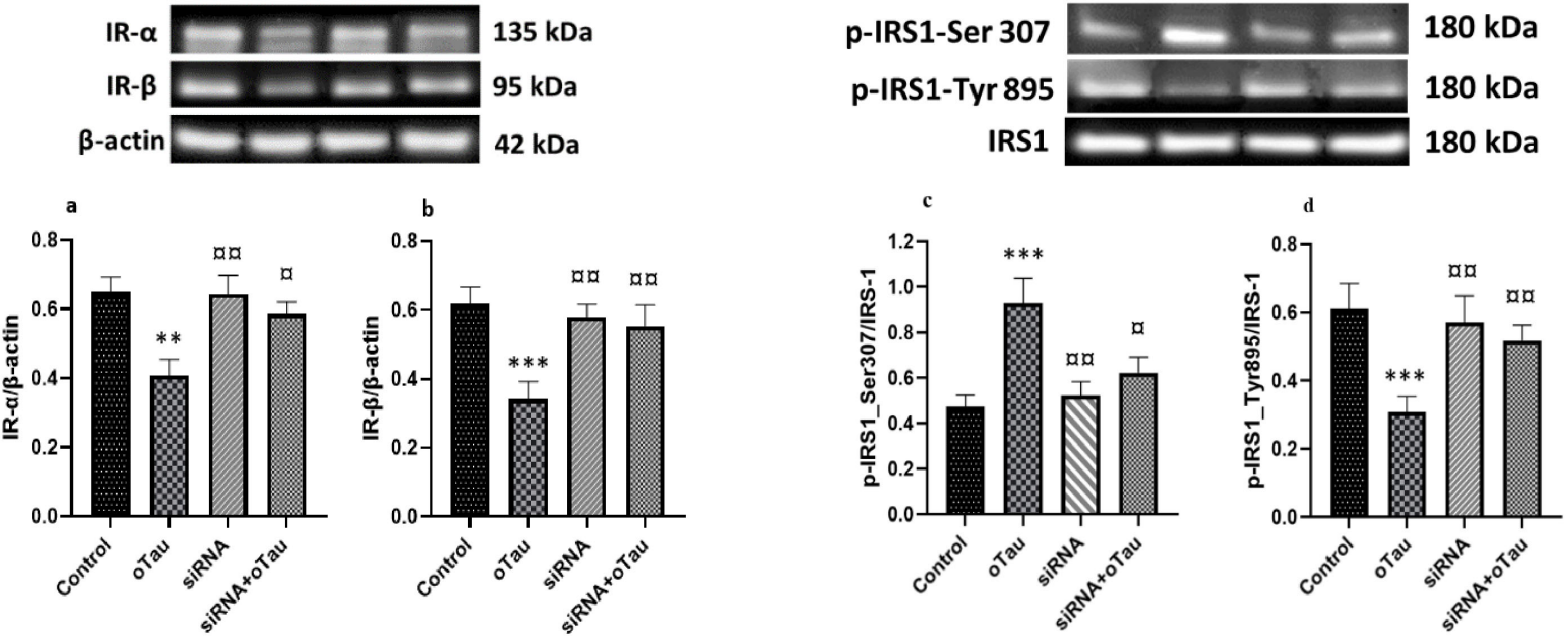
**(a)** Insulin receptor-α (IR-α) and **(b)** IR-β expression levels are significantly reduced in cerebral endothelial cells (CECs) treated with oligomeric tau (oTau) compared to the control group. **(c)** Phosphorylation of IRS-1 at the inhibitory Ser307 site is significantly increased, while **(d)** phosphorylation at the activating Tyr895 site is significantly decreased in the oTau-treated group compared to the control. However, silencing the cPLA2 gene via siRNA transfection restores the expression of both insulin receptor subunits and IRS-1 phosphorylation in oTau-exposed CECs. Data are presented as mean ± SD. Statistical significance is denoted as follows: **p < 0.01, ***p < 0.001 compared to the control group; ^¤^*p* < 0.05, ^¤¤^*p* < 0.01 compared to the oTau-treated group.

Insulin receptor-α and IR-β in CECs play an important role in insulin signaling and maintaining glucose homeostasis (Plum et al., 2005). A recent study shows that abnormal IRS-1 phosphorylation is associated with tau pathology in AD (Yarchoan et al., 2014). Several studies also indicated that hyperphosphorylated tau or tau aggregation alters endothelial cell function and disrupts insulin signaling in the brain (El Khoury et al., 2014; Mullins et al., 2017). To investigate the effect of oTau on insulin signaling via cPLA2, we exposed CECs to oTau for 24 h and found significant downregulation of both IR-α and IR-β (Figure 6). Furthermore, oTau altered the phosphorylation pattern of IRS-1, increasing phosphorylation at the inhibitory Ser307 site while decreasing phosphorylation at the activating Tyr895 site (Figure 6) –indicative of impaired insulin signaling. Notably, silencing cPLA2 by siRNA transfection effectively reversed these oTau-induced effects, restoring IR-α and IR-β expression and normalizing IRS-1 phosphorylation patterns. These findings suggest that cPLA2 plays a critical role in mediating oTau-induced insulin signaling disruption in CECs and may serve as a potential therapeutic target in tau-associated vascular insulin resistance.

## Discussion

Alzheimer’s disease and T2DM are two highly prevalent disorders worldwide (Barbagallo and Dominguez, 2014). T2DM is associated with cognitive dysfunction and an increased risk of developing neurocognitive disorders, including AD (Sims-Robinson et al., 2010; Biessels and Despa, 2018). These conditions share several pathophysiological mechanisms, such as alterations in insulin signaling, defects in glucose transporters (GLUTs), and mitochondrial dysfunction in the brain (Akter et al., 2011; Park, 2011; Shah et al., 2012; Kamal et al., 2014; Čater and Hölter, 2022). Recent studies have highlighted the role of tau pathology in both AD and T2DM, where tau-induced alterations in insulin signaling have emerged as a common pathogenic mechanism linking these two disorders and contributing to their shared pathophysiology (Rodriguez-Rodriguez et al., 2017; Gonçalves et al., 2019; El Idrissi and Alonso, 2022; Al-Lahham and Mendez, 2023; Li et al., 2023; Sarkar et al., 2023).

While numerous studies have established a strong link between tau-induced AD and T2DM, particularly through the dysfunction of insulin signaling, the mechanism(s) underlying how oTau disrupts insulin signaling in the brain have yet to be fully elucidated. This study underscores the pivotal role of cPLA2 in mediating oTau-induced disruptions in insulin signaling in CECs and 3xTg-AD mouse brain. Our findings provide novel insights into the molecular mechanisms by which oTau contributes to endothelial dysfunction and insulin resistance, suggesting the therapeutic potential of targeting the cPLA2 pathway in AD.

Cytosolic phospholipase A2 is a highly conserved enzyme widely expressed across various tissues and cell types, playing a central role in lipid signaling pathways (Leslie, 2015). Recent studies indicate that overactivation of cPLA2 has been associated with inflammation, oxidative stress, and cellular dysfunction, contributing to the pathogenesis of neurodegenerative diseases, including AD (Sun et al., 2010, 2014). Many studies have implicated cPLA2 in mediating several disease processes in the nervous system, including post-ischemic brain injury, spinal cord injury (SCI), and neurodegenerative diseases (Chen, 2012; David et al., 2012; Leslie, 2015; Sarkar and Lipinski, 2023). Additional studies also suggest that cPLA2 overactivation is involved in promoting neurodegeneration in prion diseases (Bate et al., 2010; Last et al., 2012). Our study identified significant cPLA2 activation in the cortex and hippocampus of 3xTg AD mice and oTau-treated CECs, suggesting that the involvement of cPLA2 activation in AD pathology.

Since activated cPLA2 can target and hydrolyze phospholipids in cellular membranes, releasing ARA and lysophospholipids (Sun et al., 2014), it alters the composition, structure, and physical properties of cellular membranes, leading to changes in cell function relevant to AD pathology. For example, we have previously reported that cPLA2 activated by oligomeric amyloid-β peptide (oAβ) in astrocytes targets membranes and makes them more molecularly ordered (Hicks et al., 2008). This biophysical change in cell membranes likely results from the enzymatic action of cPLA2, which hydrolyzes phospholipids to release unsaturated ARA, thereby reducing unsaturated phospholipids in the membrane and tightening their molecular packing. oAβ-activated cPLA2 also targets mitochondrial membranes in astrocytes, resulting in a loss of mitochondrial membrane potential, highlighting the role of cPLA2 activation in oAβ-induced mitochondrial dysfunction (Zhu et al., 2006). Additionally, activated cPLA2 reduces membrane-cytoskeleton connectivity, leading to increased fluid-phase macropinocytosis of soluble Aβ in microglia, suggesting a role for cPLA2 activation in microglial-mediated clearance of soluble Aβ (Teng et al., 2019). The enzymatic products of cPLA2 may also impact cellular processes significant to AD pathology. For example, exposing neural cells to ARA increases membrane fluidity, promoting α-secretase-dependent amyloid precursor protein processing (Yang et al., 2011). This study expands this line of research on the role of cPLA2 in AD pathology by demonstrating the involvement of oTau-activated cPLA2 in altering membrane function through its effect on downregulating Cav-1, a membrane protein known to stabilize lipid rafts, leading to a disruption of insulin signaling.

To date, only two published studies have reported a link between tau and phospholipase A2 (PLA2). These studies demonstrated that *in vivo* infusion of a non-specific PLA2 inhibitor, methyl arachidonyl fluorophosphonate (MAFP), in rats reduces total tau protein levels (Schaeffer et al., 2011), and that MAFP also promotes tau phosphorylation at Ser214 in primary hippocampal neurons (De-Paula et al., 2010). These studies mainly focus on the impact of activated cPLA2 on Tau expression and its phosphorylation. Since soluble oTau has been detected in extracellular spaces, we took an alternative approach and, for the first time, demonstrated the effect of oTau exposure on cPLA2 activation and its downstream impact on cellular functions, such as insulin signaling.

Under pathological conditions, tau becomes hyperphosphorylated and progressively forms aggregates ranging from small, soluble oligomeric tau species to helical filaments, and eventually to larger fibrillar aggregates that compose neurofibrillary tangles (NFTs) (Iqbal et al., 2005; Šimić et al., 2016; Pérez et al., 2018). Abnormal protein aggregation, including that of oTau, is a key hallmark of AD pathology (Muralidar et al., 2020). oTau, rather than NFTs, has been reported as the most toxic form of tau, capable of altering cellular metabolism and triggering neurodegeneration (Ghag et al., 2018). oTau is detected extracellularly in the brain and cerebrospinal fluid (CSF) of AD patients relatively early in the disease process and correlates more strongly with cognitive impairment than NFTs in mouse models of tauopathy (Sengupta et al., 2017; Ashton et al., 2022). Moreover, neuronal loss precedes the appearance of NFTs, suggesting that large fibrillar aggregates are not the primary drivers of neurodegeneration (Kuchibhotla et al., 2014). oTau is considered the principal agent responsible for spreading tau pathology between neurons throughout the brain (Gerson and Kayed, 2013; Wei et al., 2022). *In vitro* studies and tauopathy mouse models have shown that tau oligomers impair axonal transport, disrupt synaptic and mitochondrial function, and ultimately lead to neuronal death (Lasagna-Reeves et al., 2011; Reddy, 2011; Guerrero-Muñoz et al., 2015). This toxic species also accumulates in endothelial and vascular smooth muscle cells within the cerebrovasculature of AD patients and individuals with primary tauopathies such as progressive supranuclear palsy (Castillo-Carranza et al., 2017; Canepa and Fossati, 2020). The broad range of cell types affected by oTau suggests diverse pathogenic mechanisms, including neuronal dysfunction and death, neuroinflammation mediated by reactive astrocytes and microglia, and cerebrovascular dysregulation in AD and other tauopathies (Mondragón-Rodríguez et al., 2013; Alavi Naini and Soussi-Yanicostas, 2015; Kang et al., 2020; Chung et al., 2021). Furthermore, oTau has been implicated in increasing BBB permeability (Michalicova et al., 2020; Hossen et al., 2024b). Therefore, this study focuses on investigating the effects of oTau.

Recent studies indicate that pathological protein aggregates, such as oTau, significantly impact brain insulin signaling, with dysfunction in this pathway increasingly referred to as “type 3 diabetes” (Gonçalves et al., 2019; Nguyen et al., 2020; El Idrissi and Alonso, 2022). In the brain, cerebral endothelial cells play a critical role in insulin signaling by expressing insulin receptors and forming the BBB, which regulates insulin transport and signaling (Richards et al., 2010; Konishi et al., 2017; Hughes and Craft, 2022; Calon, 2023). Tau has been implicated in causing endothelial cell dysfunction and BBB disruption, both of which impair brain insulin signaling (El Idrissi and Alonso, 2022; Hussong et al., 2023; Yuan et al., 2023). However, the underlying mechanisms remain incompletely understood. Here, we demonstrate that cPLA2 activation is a key mechanism mediating oTau-induced disruption of insulin signaling.

One crucial factor contributing to impaired brain insulin signaling is altered insulin receptor activity and sensitivity (Griffith et al., 2018; Pomytkin et al., 2018). Recent studies have linked impaired insulin receptor activity to AD pathology (Folch et al., 2018; Pomytkin et al., 2018). Our findings are consistent with these studies and demonstrate significant reductions in IR-α and IR-β expression in the cerebral cortex and hippocampus of 3xTg AD mice and oTau-treated CECs. Consistently, we found that oTau exposure also reduces IR’s expressions in CECs. Importantly, IR’s expressions in oTau-exposed CECs are restored by silencing cPLA2, suggesting the role of cPLA2 in the effects of oTau on disrupting insulin signaling.

Dysregulated insulin signaling in AD has been found strongly correlated to serine/tyrosine (Ser/Tyr) phosphorylation of insulin receptor substrate1 (IRS-1) (Gual et al., 2005; Zick, 2005). Recent studies have revealed that AD brain atrophy is associated with IRS-1 expression, showing a positive relationship with IRS-1 Tyr phosphorylation and a negative relationship with IRS-1 Ser phosphorylation. IRS-1 Tyr phosphorylation activates the insulin receptor and initiates insulin signaling, whereas IRS-1 Ser phosphorylation inhibits insulin signaling (Gual et al., 2005; Draznin, 2006; Zheng and Wang, 2021). In our study, we observed alterations in IRS-1 phosphorylation, characterized by increased phosphorylation at Ser307 and decreased phosphorylation at Tyr895 in 3xTg AD mice and oTau-treated CECs, indicating a shift toward disruption of insulin signaling. Interestingly, silencing cPLA2 restored the normal phosphorylation pattern of IRS-1 in oTau-exposed CECs. Therefore, this cPLA2-mediated abnormal signaling cascade is a hallmark of impaired insulin sensitivity, linking metabolic and neurodegenerative disorders and reinforcing the connection between AD and diabetes.

Caveolin-1, a principal membrane protein of caveolae, is enriched in endothelial cells and plays a critical role in insulin receptor function by regulating receptor localization and signal transduction (Frank et al., 2003). Recent studies have shown that Cav-1 regulates insulin uptake and IR levels in endothelial cells, and that its expression is altered in T2DM mouse models (Wang et al., 2011; Shetti et al., 2023). Our findings reveal similar results, showing a significant reduction in Cav-1 expression in the cortex and hippocampus of 3xTg AD mice and oTau-treated CECs. This decrease may exacerbate insulin receptor dysfunction and downstream signaling disruptions. Importantly, we found that silencing cPLA2 restored Cav-1 expression in oTau-exposed CECs, indicating that cPLA2 activation mediates oTau-induced disruption of insulin signaling through its effects on downregulating Cav-1.

Our study suggests targeting cPLA2 be a potential therapeutic strategy for insulin signaling disruption in the brain. A recent study found that cPLA2 knockout (KO) mice can protect brains from post-ischemic brain injury induced by middle cerebral artery occlusion (Zheng and Wang, 2021). The KO mice also exhibit fewer neurological deficits compared with WT mice. Another study found that inhibiting cPLA2 (AACOCF3) or genetically ablating cPLA2 (cPLA2 KO C57Bl/6 mice) results in improved motor deficiencies and less tissue damage from SCI (Liu et al., 2014). Our study found that silencing cPLA2 by siRNA transfection effectively mitigated the detrimental effects of oTau on insulin signaling, restoring normal IR-α and IR-β expression, IRS-1 phosphorylation patterns, and Cav-1 levels. These findings highlight cPLA2 as a critical contributor to endothelial dysfunction and suggest its inhibition as a promising therapeutic strategy.

**FIGURE 7:**
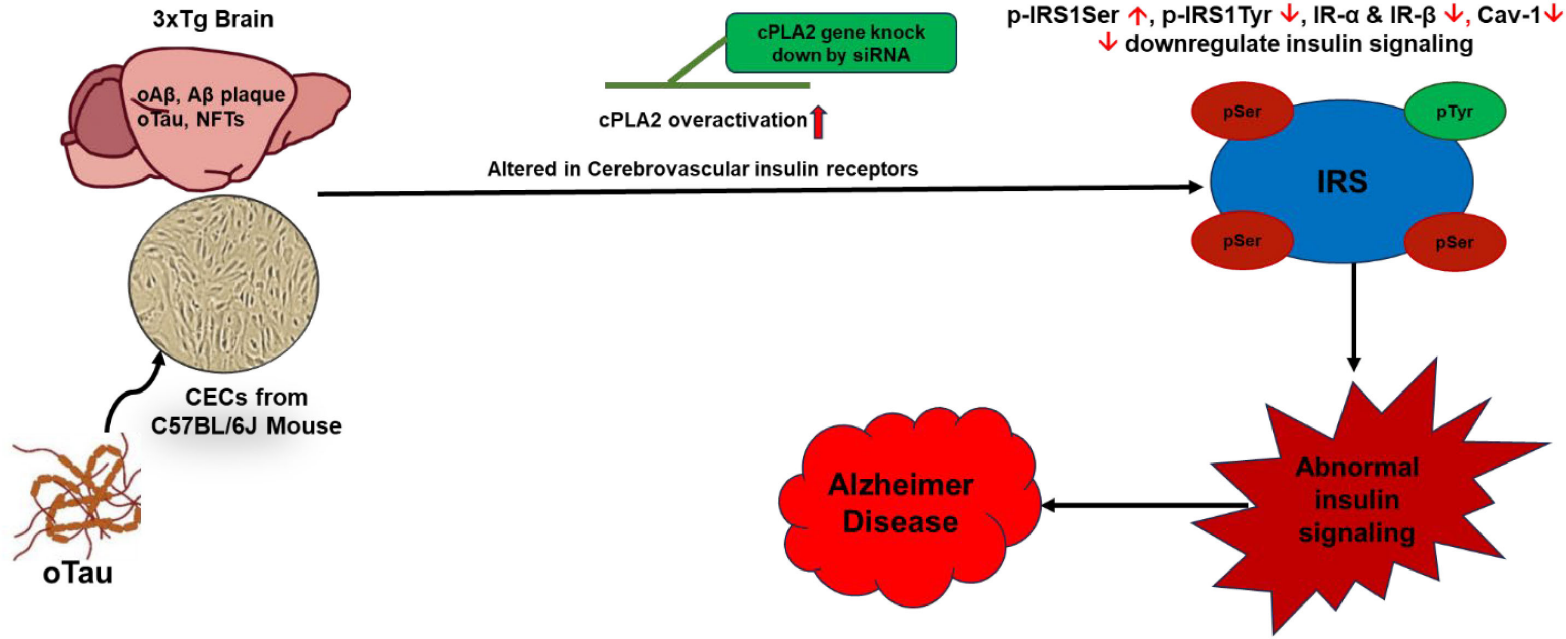
Oligomeric tau (oTau)-induced abnormalities in insulin signaling in CECs and the 3xTg AD mouse brain is mediated through cPLA2 pathway. oTau induces overactivation of the cPLA2, disrupts CEC function, and alters insulin signaling. Targeting the cPLA2 pathway could serve as a potential therapeutic strategy to restore insulin signaling in CECs in the mouse brain. IR, insulin receptor; IRS, insulin receptor substrates.

In summary, our findings suggest that cPLA2 plays a central role in oTau-induced abnormalities in insulin signaling and endothelial dysfunction in the brain. Targeting cPLA2 presents a promising therapeutic approach for restoring insulin signaling and mitigating vascular and metabolic abnormalities in AD (Figure 7). This study contributes to the growing understanding of the molecular mechanisms linking neurodegeneration, endothelial dysfunction, and metabolic dysregulation, paving the way for novel therapeutic interventions in AD.

## Data Availability

The original contributions presented in this study are included in this article/Supplementary material, further inquiries can be directed to the corresponding author.
