# Supplementary material for: Cytosolic phospholipase A2 links tau pathology to insulin signaling impairment in Alzheimer’s disease
Source: Front Aging Neurosci. 2025 Nov 7;17:1671986. doi: 10.3389/fnagi.2025.1671986 (PMC12634615; doi:10.3389/fnagi.2025.1671986)
Supplement: Supplementary file 1 [file Data_Sheet_1.pdf]

Cytosolic Phospholipase A2 links Tau Pathology to Insulin Signaling Impairment in Alzheimer's Disease

Faruk Hossen[1], Javier Hung[1], Hamza Odeh[1], Grace Y. Sun[2], James C. Lee[1*]

[1]Richard and Loan Hill Department of Biomedical Engineering, University of Illinois at Chicago, Chicago, IL 60607, United States

[2]Department of Biochemistry, University of Missouri, Columbia, MO 65211, United States

***Correspondence**: LeeJam@uic.edu

Supplementary Table 1: List of Antibodies

Antibody	Manufacturer	Catalog number
IR-α	Cell Signaling	74118
IR-β	Cell Signaling	3025
IRS-1	Cell Signaling	2382
Phospho-IRS-1 (Ser307)	Cell Signaling	2381
Phospho-IRS-1 (Tyr895)	Cell Signaling	3070
Caveolin-1	Cell Signaling	3267
cPLA2	Cell Signaling	2832
Phospho-cPLA2	Cell Signaling	2831
Anti-Tau (T22)	Sigma	ABN454-I

Supplementary Table 2: List of kits and Reagents

Assay kits/reagents	Manufacturer	Catalog number
BCA protein assay kit	Thermo Scientific™	23227
Tau-441	rPeptide	T-1001-2
Heparin	STEMCELL Technologies	07980
EGM™-2 MV BulletKit™	Lonza	CC-3202
Puromycin	Sigma	P8833
Lipofectamine™ 3000 Transfection Reagent	Invitrogen™	L3000001
Silencer Select Pre-designed siRNA (Pla2g4a)	Ambion (Thermo fisher)	4390771
Universal scrambled negative control siRNA	OriGene Technologies, Inc.	SR30004

Sequence of siRNA

The sequences of the siRNA targeting cPLA2 are below:

Ambion Silencer Select pre-designed siRNA targeting Pla2g4a (catalog #4390771)

Sense GAGCUGAUGUUUGCCGAUUTT

Antise AAUCGGCAAACAUCAGCUCTG

Supplementary Fig.1

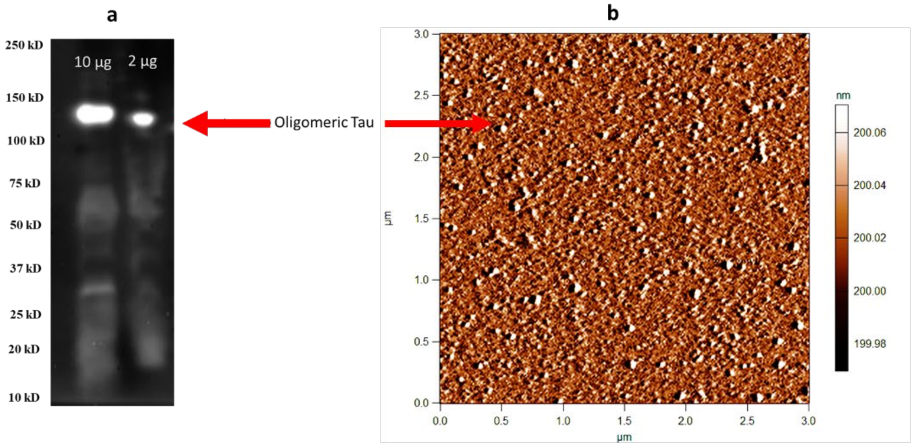

Supplementary Fig. 1: Characterization of oligomeric Tau (oTau) formation using Western blot analysis **(a)** and atomic force microscopy (AFM) **(b)**. oTau species are >50 kDa, typically ranging between 75–150 kDa. Western blot analysis was performed using the anti-Tau (T-22) antibody, which specifically recognizes oTau and does not show significant reactivity with its monomeric or fibrillar forms. Oligomeric Tau preparations (panel b) were prepared as described in the Materials and Methods section and diluted 1:10 before AFM analysis.
